# Evaluation of an oral joint supplement on gait kinematics and biomarkers of cartilage metabolism and inflammation in mature riding horses

**DOI:** 10.1093/tas/txaa150

**Published:** 2020-08-24

**Authors:** Mattea L Much, Jessica L Leatherwood, Rafael E Martinez, Brittany L Silvers, Casey F Basta, Lydia F Gray, Amanda N Bradbery

**Affiliations:** 1 Texas A&M University, Department of Animal Science, College Station, TX; 2 SmartPak Equine LLC, Plymouth, MA

**Keywords:** collagen, equine, gait, inflammation, nutraceutical, osteoarthritis

## Abstract

Twenty stock-type horses (589 ± 126 kg BW; 13 ± 8 yr) were used in a completely randomized design for 28-d to evaluate the impact of a joint supplement on gait kinematics, inflammation, and cartilage metabolism. Horses were stratified by age, sex, body weight (BW), and initial lameness scores and were randomly assigned to one of two dietary treatments consisting of either a 100-g placebo top-dressed daily to 0.6% BW (as-fed) commercial concentrate (CON; *n* = 10; SafeChoice Original, Cargill, Inc.), or an oral joint supplement (SmartPak Equine LLC) containing glucosamine, chondroitin sulfate, hyaluronic acid, methylsulfonylmethane, turmeric, resveratrol, collagen, silica, and boron (TRT; *n* = 10). Horses were group-housed with ad libitum access to coastal bermudagrass hay (*Cynodon dactylon*) and allowed to graze pasture 2 h/d. Horses were exercised progressively 4 d/wk at 45 min each. On days 13 and 27, blood was harvested followed by a 19.3-km exercise stressor on concrete. Horses traveled at the walk, with no more than 15 min at the trot. Every 14 d, BW and BCS were recorded, and blood was collected for plasma prostaglandin E_2_ (PGE_2_), serum collagenase cleavage neopeptide (C2C), carboxypropeptide of type II collagen (CPII), and chondroitin sulfate 846 epitope (CS846) analysis. Kinematic gait analysis was performed every 14 d (Kinovea v.0.8.15) to determine stride length (SL) and range of motion (ROM) of the knee and hock at the walk and trot. Data were analyzed using PROC MIXED of SAS. All horses increased BW and BCS over time (*P* ≤ 0.01). Hock ROM increased in TRT horses (*P* ≤ 0.02) at the walk and tended to increase at the trot compared to CON (*P* = 0.09). At the walk, SL and knee ROM increased over time, independent of dietary treatment (*P* ≤ 0.01); no time effect was observed at the trot (*P* > 0.15). Regardless of treatment, C2C and CPII increased over time (*P* ≤ 0.05) and no effect was observed for CS846 or PGE_2_ (*P* > 0.12). In response to the exercise stressor, CPII and PGE_2_ decreased (*P* ≤ 0.05) from day 13 to 14, and CS846 and PGE_2_ tended to decrease (*P ≤* 0.10) from day 27 to 28, independent of dietary treatment. In conclusion, hock ROM at the walk and trot was most sensitive to dietary treatment. Supplementation did not alter biomarker concentration of collagen metabolites or systemic inflammation in the 28-d period, but a future study utilizing arthrocentesis may be warranted to specifically evaluate intra-articular response to dietary treatment.

## INTRODUCTION

Natural, repetitive concussion of the lower limbs is suggested to contribute to the early development and progression of osteoarthritis (**OA**) in performance horses (Cantley et al., 1999). Although no proven method of prevention exists, prior research suggests that targeted dietary supplements may support joint health to preserve the integrity of articular cartilage and longevity of the exercising horse (Hanson et al., 1997; Forsyth et al., 2006; Leatherwood et al., 2016). Previous trials investigating the use of oral joint nutraceuticals in horses have conflicting results that are challenging to compare, producing skepticism regarding the benefit of feeding these products to performance horses.

Articulating joints are comprised of collagenous protein structures that rely on homeostatic turnover to maintain optimal function. Type II cartilage is prevalent in joints where catabolic collagen cleavage neoepitope (**C2C**), anabolic carboxypropeptide of type II collagen (**CPII**), and aggrecan chondroitin sulfate 846 (**CS846)** epitope biomarkers can be used to monitor the effect of exercise and progression of OA in both serum and synovial fluid (Frisbie et al., 2008; McIlwraith, 2008; Garvican et al., 2010). Prostaglandin E_2_ (**PGE**_**2**_) can also be used as a marker for OA because its concentration increases in affected cartilage and is associated with disease pathogenesis (Abramson, 2008; Attur et al. 2012). After collagen protein is catabolized intra-articularly, biomarkers diffuse through the synovium and can be detected in circulation (Ray et al., 1996). These biomarkers provide an objective and quantitative approach to identifying metabolic alterations in articular cartilage and to evaluate the impact of potential dietary therapies (McIlwraith, 2005).

Consistent objective means to evaluate lameness in horses is limited; however, kinematic analysis software may enhance the reliability of the evaluator’s diagnosis (Hewetson et al., 2006). This method disregards external forces affecting movement, allowing individual joints to be studied quantitatively and noninvasively while enhancing the understanding of locomotion (Clayton, 1991a; Peham et al., 2001). Gait analysis software may be used to evaluate the potential efficacy of joint health nutraceuticals intended to reduce inflammation (Coverdale and Campbell, 2015). Therefore, the objectives of this study were to determine the influence of an oral joint supplement on gait kinematics, systemic inflammation, and markers of cartilage metabolism in mature horses undergoing light exercise.

## MATERIALS AND METHODS

The experimental protocol was approved by the Institutional Animal Care and Use Committee at Texas A&M University (AUP# 2019-0228).

### Horses and Dietary Treatments

Twenty stock-type horses (589 ± 126 kg BW; 13 ± 8 yr) from a previously established herd at Texas A&M University (College Station, TX) were utilized in a completely randomized design for a 28-d trial. Horses were stratified by body weight (**BW**), age, sex, and initial lameness scores and were randomly assigned to receive one of two dietary treatments that consisted of horses receiving 100 g of an inactive placebo of dehydrated alfalfa meal, top-dressed once daily (**CON**; *n* = 10), or 100 g of a commercially available oral joint supplement (SmartPak Equine, LLC, Plymouth, MA) that was formulated to include 15,000-mg hydrolyzed collagen, 8,000-mg turmeric root powder, 7,500-mg methylsulfonylmethane (**MSM**), 5,000-mg glucosamine HCl, 1,000-mg resveratrol, 500-mg chondroitin sulfate, 250-mg silica, 100-mg sodium hyaluronate (**HA**), and 25-mg boron (**TRT**; *n* = 10). Investigators were blinded to dietary treatments through completion of data analysis, with CON and TRT supplements preweighed and alphabetically coded.

Prior to the start of the trial, lameness evaluations were performed by a licensed veterinarian (Equine Field Service, Texas A&M University, College Station, TX) using a scale of 0 to 5 outlined by the American Association of Equine Practitioners (AAEP, 2019). Horses selected for the trial were determined to be either free of lameness (Score 0; *n* = 18) or had lameness that was difficult to observe or not consistently apparent at the walk or trot (Score 1; *n* = 2; AAEP, 2019). All horses were offered 0.6% BW (as-fed) of a commercially available concentrate (SafeChoice Original, Cargill Inc.) daily to meet or slightly exceed the nutritional requirements for mature horses undergoing light exercise (NRC, 2007). This ration was divided evenly between two meals offered at 12-h intervals using attachable feed bags (Derby Originals LLC; North Canton, OH). Horses were allowed 60 min to consume concentrate meals and intakes and orts were recorded daily. Horses had ad libitum access to water and round bales of coastal bermudagrass (*Cynodon dactylon*) hay in a group-housed setting and all horses grazed 2 h/d on coastal bermudagrass pasture. Composited samples of concentrate, hay, and pasture were analyzed for nutrient content using a commercial laboratory (Equi-Analytical Laboratories, Ithaca, NY; Table 1).

**Table 1.** Nutrient composition of pelleted concentrate, Coastal bermudagrass (*Cynodon dactylon*) hay, and Coastal bermudagrass pasture fed to mature stock-type horses

Item^*a*^	Concentrate^*b*^	Hay	Pasture
Nutrient, % DM			
CP	16.5	6.5	8.2
Starch	22.7	5.0	6.4
ADF	15.2	37.7	35.1
NDF	28.2	72.0	66.4
Crude Fat	7.7	1.7	1.7

^*a*^Equi-Analytical Laboratories (Ithaca, NY).

^*b*^Concentrate consisted of 0.6% BW (as-fed) daily of a commercially formulated concentrate (Safe Choice Original, Cargill, Inc.)

Every 14 d, BW was recorded using a calibrated digital platform scale (Bastrop Scale Inc., Bastrop, TX) and individual intakes of concentrate were adjusted accordingly. Three independent and trained observers obtained BCS every 14 d and an average value was recorded. Anti-inflammatory medications were withheld for a minimum of 4 wk prior to and during the experimental period, and all farrier work was completed by the same professional farrier at 4-wk intervals.

### Exercise

Four days/week, horses were ridden for 45 min as part of the training program at Parsons Mounted Cavalry (Texas A&M Corps of Cadets, College Station, TX). Horses had been similarly exercised for the previous 4 wk leading up to the start of the trial. During the study, horses were exercised on a natural dirt surface in accordance with course requirements, and activities progressed collectively as a group. Course topics included a transition from walk-trot drills through wk 2, to introducing the canter beginning at wk 3 (Table 2).

**Table 2.** Weekly ridden exercise summary for horses during the 28-d trial

Item^*a*^	Walk	Trot	Canter
Total time (min)			
Week 1	35	10	–
Week 2	25	20	–
Week 3	20	20	5
Week 4	20	15	10

^a^Horses were exercised 4 d/wk in class at Parsons Mounted Cavalry (Texas A&M Corps of Cadets). Workload intensity steadily increased throughout the trial and focused on drills at the walk, trot, and canter.

### Exercise Stressor

On days 13 and 27 of the study, all horses participated in a 6-h exercise stressor, traveling a total of 19.3-km on a parade route to evaluate the physiological response to increased exercise duration and load on a concrete surface (Tessutti et al., 2012). Both days encompassed the groups’ mounted departure from the equine facility, riding on public roads traveling predominantly at the walk, and for no more than 15 min at the trot, to the Texas A&M University campus. Upon arrival, the unit traveled through campus and conducted three practice laps (402 m each) around the football stadium followed by a 2-h rest period in which horses were offered hay and water. Horses then moved as a unit around the track inside the football stadium (402 m) that was followed by return to the equine facility along the same route.

### Sample Collection and Laboratory Analysis

Plasma and serum samples were collected via jugular venipuncture on days 0, 13, 14, 27, and 28, prior to morning feeding. Samples intended for serum analysis were collected into 10-mL evacuated nonadditive tubes (BD Vacutainer, Franklin Lakes, NJ) and allowed to remain at room temperature to clot for 30 min prior to processing. Samples intended for plasma analysis were collected into 7.5-mL evacuated tubes containing 0.081 mL of 15% K_3_ EDTA solution and 12.15 mg K_3_ (BD Vacutainer, Franklin Lakes, NJ) and were immediately stored on ice. All samples were centrifuged at 2,000 × *g* at 4 °C for 20 min and the supernatant was aliquoted and then stored in 1.5-mL microcentrifuge tubes at −80 °C prior to analysis.

Samples were analyzed in duplicate using commercially available ELISA kits, previously validated for use in horses, to determine plasma PGE_2_ (R&D Systems, Inc., Minneapolis, MN), and serum biomarkers related to cartilage metabolism that included C2C, CPII, and CS846 (IBEX Pharmaceuticals, Inc., Montreal, QC, Canada). Dilutions were made with diluents provided by the kit before beginning the assay. Standards were diluted 1:3, 1:5, 1:10, and 1:5 for PGE_2_, C2C, CPII, and CS846, respectively. Minimum detectible limits for C2C, CPII, and CS846 were 10, 50, and 20 ng/mL, respectively. Intra-assay precision for C2C and CPII ranged from 0.30% to 9.50% and 0% and 7.80%, whereas the inter-assay CV was ≤7.98%. Intra-assay CV for the CS846 epitope ranged from 0.60% to 9.40% and maximum inter-assay was 9.06%. The PGE_2_ intra-assay CV ranged between 0.30% and 9.90% and an inter-assay CV of 7.57%. All plates were read using a microplate reader (BioRad 680 Microplate Reader, BioRad Laboratories, Hercules, CA).

### Gait Kinematic Analysis

Gait kinematic analysis was conducted on days 0, 14, and 28 of the study. All horses were led by the same handler along a 10-m path on a solid dirt surface three times each at both the walk and trot. Square, 2.5-cm adhesive markers were used to mark joints of interest and were placed by a single individual on the right forelimb and hindlimb to aid in visibility and calibration. The distance measured between the greater tubercle of the humerus and the ulnar carpal bone was recorded using a soft tape measure and used for software calibration. The forelimb markers included six locations: the greater tubercle of the humerus, lateral humeral epicondyle, ulnar carpal bones, lateral metacarpal epicondyle, middle phalanx–proximal phalanx junction, and proximal phalanx–distal phalanx junction. The hindlimb markers included five locations: the distal phalanx–middle phalanx junction, middle phalanx–proximal phalanx junction, proximal phalanx–third metacarpal junction, tarsal bones, and lateral femoral epicondyle.

Video recordings of each horse were made using an iPad Pro (Apple, CA, 11”) placed on a tripod set at a height of 142 cm to the camera lens and 9.14 m from the center of the measured pathway. Gait analysis was performed by an individual researcher using Kinovea software v. 0.8.15, a two-dimensional motion analysis program to assess stride length (**SL**) and range of motion (**ROM**) for the knee and hock. An SL was defined as the distance from the initial point of contact of the front right limb’s stance phase-1 to the next point of contact after this limb completed one swing phase and subsequently entered stance phase-2 (Clayton and van Weeren, 2013). Angular joint ROM was defined as the difference between the maximum and minimum angles achieved by that joint during one stride (Morales et al., 1998). To account for biological variability, each horse was evaluated three times at each gait and the average of the three was used to determine SL and ROM. Velocity was also determined using the average of three time points per horse for each sample day, calculated from video recordings using the time taken to cover a given distance (Morales et al., 1998).

### Statistical Analysis

All data were analyzed using PROC MIXED in SAS (SAS Institute Inc., Cary, NC). The model contained fixed effects for treatment, time, and treatment × time interactions. Age was examined in the model and no effect was observed; therefore, it was removed from the model statement to conserve degrees of freedom. Data were tested for normality using PROC UNIVARIATE prior to analysis and all non-normal data (PGE_2_, CPII, and CS846) were log-transformed to achieve normality. Log-transformed data are presented as log_10_ LSMeans ± SEM and untransformed data are presented as LSMeans ± SEM. Outliers were identified using box plots of the residuals and were removed if 2SD from the mean. Biomarker data from the exercise stressor required a covariate structure using data obtained on day 0, and the model included a random effect of horse within treatment to account for individual variability. Delta values were defined as the change between days 0 and 28. Significance was declared at *P* ≤ 0.05 and a trend toward significance was declared at *P* ≤ 0.10.

## RESULTS

### Intake and Performance Characteristics

Horses readily consumed concentrate and no refusals were recorded throughout the 28-d trial. Mean BW and BCS did not differ between dietary treatments (*P* = 0.20); however, all horses increased (*P* ≤ 0.01) in BW from 566.11 ± 16.28 to 574.5 ± 16.28 kg (Table 3) and BCS increased from 6.1 ± 0.18 to 6.29 ± 0.18 regardless of dietary treatment (*P* = 0.03; Table 3).

**Table 3.** Mean change in body weight (BW) and body condition score (BCS) in horses receiving a pelleted concentrate and supplemented with either 100 g of an inactive placebo top-dressed once daily (n = 10; CON), or an active-ingredient oral joint supplement (*n* = 10; TRT)

	Treatment^*a*^			
Item	TRT	CON	SEM	*P-*value^*b*^
Mean BW, kg				
Day 0	575.2	560.19	32.56	0.58
Day 14	589.53	570.22	32.56	0.46
Day 28	587.54	566.33	32.56	0.43
BCS (Scale 1–9)				
Day 0	6.23	6.04	0.36	0.46
Day 14	6.02	6.04	0.36	0.96
Day 28	6.45	6.15	0.36	0.39

^*a*^Treatments consisted of either 100 g of an inactive placebo (CON) top-dressed once daily, or an active-ingredient oral joint supplement containing glucosamine, chondroitin sulfate, hyaluronic acid, methylsulfonylmethane, turmeric, resveratrol, collagen, silica, and boron (TRT).

^*b*^Main effect of dietary treatment.

### Markers of Cartilage Metabolism and Inflammation

There was no influence of dietary treatment on serum C2C or CPII (Table 4; *P* = 0.54 and *P* = 0.45, respectively); however, both increased over time (*P* ≤ 0.05) in response to increasing exercise load. Throughout the trial, overall C2C and Log_10_ CPII concentration increased between days 0 and 28 (186.63 to 214.06 ± 8.24and 3.45 to 3.51 ± 0.02 ng/mL, respectively). No effect of dietary treatment was observed for serum CS846 concentrations, indicative of aggrecan turnover (*P* = 0.12; Table 4); however, horses on TRT tended (*P* = 0.08; Table 3) to have a larger change in Log_10_ CS846 concentration (0.04 ± 0.05 ng/mL) compared with CON horses (-0.09 ± 0.05). No effects of dietary treatment or time were observed for plasma PGE_2_ concentrations (*P ≥* 0.15; Table 4).

**Table 4.** Mean change in serum and plasma biomarkers in horses receiving a pelleted concentrate and supplemented with either 100 g of an inactive placebo top-dressed once daily (CON), or an active-ingredient oral joint supplement (TRT)

	Treatment^*a*^			
Item	TRT	CON	SEM	*P-*value^*b*^
C2C, ng/mL^*c*^				
Day 0	184.78	188.47	16.47	0.65
Day 14	198.97	213.77	16.47	0.54
Day 28	213.60	214.51	16.47	0.93
∆ 28–0	28.82	23.01	17.42	0.82
Log_10_ CPII, ng/mL^*c*^				
Day 0	3.48	3.42	0.04	0.19
Day 14	3.48	3.47	0.04	0.45
Day 28	3.52	3.50	0.02	0.69
∆ 28-0	0.04	0.07	0.04	0.54
Log_10_CS846, ng/mL^*d*^				
Day 0	2.67	2.65	0.14	0.87
Day 14	2.71	2.65	0.14	0.57
Day 28	2.72	2.56	0.15	0.29
∆ 28–0	0.04^*f*^	−0.09^*g*^	0.07	0.08
Log_10_PGE_2_, pg/mL^*e*^				
Day 0	2.90	3.13	0.19	0.24
Day 14	2.88	3.18	0.19	0.15
Day 28	2.91	3.18	0.19	0.17
∆ 28–0	0.01	−0.12	0.20	0.49

^*a*^Treatments consisted of either 100 g of an inactive placebo (CON) top-dressed once daily, or an active-ingredient oral joint supplement containing glucosamine, chondroitin sulfate, hyaluronic acid, methylsulfonylmethane, turmeric, resveratrol, collagen, silica, and boron (TRT).

^*b*^Main effect of dietary treatment.

^*c*^TRT (*n* = 10); CON (*n* = 9).

^*d*^TRT (*n* = 9); CON (n = 9).

^*e*^TRT (*n* = 10); CON (*n* = 7).

^*f,g*^ Within row, superscripts indicate tendency toward dietary treatment difference *P* ≤ 0.10.

### Exercise Stressor

No treatment × time effects were observed in response to either exercise stressor performed on days 13 and 27 (*P* ≥ 0.14; Table 5) of the study; therefore, the time effect is reported to illustrate differences over time in response to the exercise stressor. Markers including CPII and PGE_2_ decreased (*P* ≤ 0.05; Table 5) in all horses, regardless of diet, on day 14 following the initial exercise stressor conducted on day 13. Concentrations of CS846 and PGE_2_ also tended (*P* ≤ 0.10; Table 5) to decline on day 28 in all horses following the final exercise stressor that occurred on day 27 of the experiment.

**Table 5.** The serum concentration (ng/mL) for biomarkers of collagen turnover and plasma concentration (pg/mL) for a biomarker of systemic inflammation over time in horses after exercise stressors conducted on days 13 and 27 in mature riding horses

	Day				Day			
Item	13	14	SEM	*P-*value^*a*^	27	28	SEM	*P-*value^*b*^
C2C, ng/mL^*b*^	197.89	206.48	11.9	0.48	200.09	214.00	10.97	0.22
CPII, ng/mL^*c*^	3417.01^*h*^	3052.30^*i*^	168.49	0.05	3579.34	3306.88	231.69	0.26
CS846, ng/mL^*d*,*e*^	665.00	561.75	64.86	0.13	650.62^*h*^	529.94^*i*^	61.11	0.07
PGE_2_, pg/mL^*f*,*g*^	2016.79	1521.72	139.56	0.002	1558.21^*h*^	1269.09^*i*^	145.78	0.07

Treatments consisted of either 100 g of an inactive placebo (CON) top-dressed once daily, or an active-ingredient oral joint supplement containing glucosamine, chondroitin sulfate, hyaluronic acid, methylsulfonylmethane, turmeric, resveratrol, collagen, silica, and boron (TRT).

^*a*^Main effect of day. There was no effect of dietary treatment or treatment × day.

^*b*^TRT (*n* = 9); CON; days 13, 14 and days 27, 28.

^*c*^TRT (*n* = 10); CON (*n* = 9); days 13, 14 and days 27, 28.

^*d*^TRT (*n* = 10); CON (*n* = 9); days 13, 14.

^*e*^TRT (*n* = 9); CON (*n* = 9); days 27, 28.

^*f*^TRT (*n* = 10); CON (*n* = 8); days 13, 14.

^*g*^TRT (*n* = 10); CON (*n* = 7); days 27, 28.

^*h,i*^Within row, superscripts indicate tendency toward dietary treatment difference *P* ≤ 0.10.

### Gait Kinematics

#### Stride length.

To account for intravariation in horse velocity, an average of three timepoints for velocity were analyzed for each horse per sample day. No treatment × time difference in velocity was detected at the walk or trot (*P* = 0.96; data not shown). While evaluating the walk, SL increased (*P* ≤ 0.01; Table 6) over time but did not differ between dietary treatments (*P* = 0.78) as values increased from day 0 to 28 (140.66 ± 3.09 to 185.58 ± 3.09 cm, respectively). Dietary treatment did not affect SL at the trot (*P* = 0.15; Table 6).

**Table 6.** Mean change in gait variables of interest including SL and ROM in horses receiving a pelleted concentrate and supplemented with either 100 g of an inactive placebo top-dressed once daily (CON), or an active-ingredient oral joint supplement (TRT)

	Treatment^*a*^			
Item	TRT	CON	SEM	*P-*value^*b*^
ROM, degrees, Knee-Walk				
Day 0	54.53	55.07	3.09	0.87
Day 14	56.40	58.27	3.05	0.54
Day 28	67.90	68.63	3.09	0.98
∆ 28–0	13.37	13.93	2.56	0.88
ROM, degrees, Hock-Walk				
Day 0	29.40	31.67	2.22	0.38
Day 14	30.97	28.41	2.15	0.30
Day 28	46.87^*e*^	39.70^*f*^	2.22	<0.01
∆ 28–0	17.47^*e*^	7.55^*f*^	2.92	0.03
SL, cm, Walk				
Day 0	139.80	140.57	6.43	0.91
Day 14	141.64	143.66	6.19	0.57
Day 28	188.60	181.81	6.43	0.30
∆ 28–0	48.81	41.25	6.12	0.38
ROM, degrees, Knee-Trot^*c*^				
Day 0	68.10	67.67	2.90	0.88
Day 14	69.00	69.00	2.70	0.82
Day 28	67.83	70.22	2.90	0.42
∆ 28–0	−0.27	2.56	2.08	0.34
ROM, degrees, Hock-Trot^*d*^				
Day 0	42.30	39.78	2.20	0.26
Day 14	44.40	42.59	2.23	0.84
Day 28	46.87^*e*^	42.93^*f*^	2.20	0.08
∆ 28–0	4.57	3.15	2.10	0.63
SL, cm, Trot				
Day 0	189.24	191.07	8.23	0.83
Day 14	193.42	183.54	7.88	0.33
Day 28	187.72	192.89	8.23	0.53
∆ 28–0	−1.52	1.82	6.91	0.73

^*a*^Treatments consisted of either 100 g of an inactive placebo (CON) top-dressed once daily, or an active-ingredient oral joint supplement containing glucosamine, chondroitin sulfate, hyaluronic acid, methylsulfonylmethane, turmeric, resveratrol, collagen, silica, and boron (TRT).

^*b*^Main effect of dietary treatment.

^*c*^TRT (*n* = 10); CON (*n* = 9).

^*d*^TRT (*n* = 9); CON (*n* = 10).

^*e*,*f*^ Within row, superscripts indicate tendency toward a dietary treatment difference *P* ≤ 0.10.

#### Range of motion.

There was no effect of treatment for knee ROM at the walk or trot (*P* = 0.48); however, there was an effect of time at the walk (*P* ≤ 0.01; Table 6). Knee ROM on day 0 averaged 54.8 ± 1.53° and increased to 68.23 ± 1.53° on day 28. Regarding hock ROM, a treatment × time interaction was observed at the walk (*P* = 0.01; Table 6) as TRT horses had a greater ROM (46.78 ± 1.56°) on day 28 compared with CON (39.79 ± 1.56°). When deltas were calculated, TRT horses also displayed a greater change in hock ROM (17.47 ± 2.92°) at the walk compared with CON horses (*P* = 0.03; Table 6). An effect of time (*P* = 0.03) was observed for hock ROM at the trot with both groups increasing ROM throughout the trial. Additionally, TRT horses tended to have a higher ROM (44.87 ± 1.51°) than CON horses (42.93 ± 1.60°; *P* = 0.09; Table 6).

## DISCUSSION

This study assessed the impact of a multi-ingredient oral joint supplement on mature, lightly exercised horses in response to weekly exercise and two performance events. Throughout the study, average BW and BCS increased across both dietary treatments, which may be attributed to the transition from group feeding to individual feeding at the start of the trial period. Basal diets met the recommended DE requirement for mature horses undergoing light exercise (24.0 Mcal/d; NRC, 2007), with TRT horses receiving 26.75 Mcal/d and CON horses receiving 26.06 Mcal/d. The DE values were determined by calculating the estimated dry-matter forage intake of 2% BW/d in addition to the provided average dry-matter concentrate ration provided daily.

In the current study, SL at the walk and trot were not influenced by dietary treatment. Stride length may be influenced by several extraneous factors, such as track surface, velocity, and pain (Yamanobe et al., 1993; Galisteo et al., 1997; Chateau et al., 2010). To minimize extraneous factors that could affect SL, horses in the current study were walked and trotted on the same surface by the same experienced handler at each sample timepoint. In a previous study evaluating the effect of oral supplementation of glucosamine-chondroitin sulfate in horses diagnosed with degenerative joint disease, investigators observed a significant increase in SL at the walk from 165 to 177 cm during the first 2 wk of supplementation, which was followed by a plateau for the remainder of the 6 wk trial (Hanson et al., 1997). In the current study, a plateau in SL at the walk was observed during the first 2 wk of supplementation, and an increase in SL across both treatments occurred during the last 2 wk of the trial. Reducing inflammation, and presumably pain, in response to feeding a joint supplement, has been associated with increased SL and improved locomotor symmetry in the horse (Clayton et al., 2002; Woodward et al., 2005). Forsyth et al. (2006) provided oral supplementation of glucosamine hydrochloride and CS to a group of horses for 12 wk, assessing joint ROM and SL using two-dimentional kinematic software every 4 wk. Starting at week 8, researchers observed improved ROM at the elbow, stifle, and rear fetlock, and increased SL in horses receiving the supplement. These results suggest that the possibility for more robust improvement in gait parameters of horses in the current study should they have undergone a longer supplementation period.

Knee ROM at the walk and trot increased over time but did not differ by dietary treatment. Joint motion is affected by conformation, intra-articular composition, and extra-articular support systems (McIlwraith, 1996). Internal joint structures should enable a healthy horse to move smoothly with minimal friction due to the low viscosity of synovial fluid that prevents fusion of articular material and lowers surface tension (Palmer and Bertone, 1996). In the current study, the population of horses selected was scored ≤1 following a preliminary lameness exam, meaning they did not show any clinical sign of lameness or signs were difficult to observe and inconsistent (AAEP, 2019). Horses were exercised with a progressive training load for each of the 4 wk, including a transition from walk/trot drills through wk 2, to introducing the canter beginning at wk 3. It is possible that the increase in knee ROM observed over time across both groups was due to dynamic suppling, an effect of progressive weight bearing exercise involving eccentric and concentric cycles of muscle contraction (Clayton, 1991b; Godges et al., 1993).

Stretching joints through the use of extensor muscles can promote enhanced locomotor effects. In a previous gait analysis trial, researchers also observed an increase in stride length and knee range of motion at the trot over a 28-d period for horses in a control group that were exercised similarly to horses in the current trial (Coverdale and Campbell, 2014). In the current study, horses on TRT showed greater hock ROM at the walk throughout the trial, and a tendency for increased hock ROM at the trot, in response to dietary treatment. Although limited research has been conducted with nutraceutical intervention specific to improving hock function, data from the current study indicate that this region may be sensitive to ingredients provided in the oral supplement.

It is challenging to correlate the increased hock ROM in the current study to a specific ingredient within the supplement used in the current study; however, the combination of ingredients likely had a multifactorial influence in intra- and extra-articular structures to improve joint fluidity and ROM at the walk and trot. The bioavailability of these nutraceutical compounds and mechanism for incorporation into targeted intra-articular joints is largely unknown. Evaluating serum and plasma concentrations of specific ingredients would be useful in understanding the absorption rate of the active ingredients in the supplement.

Horses with intra-articular inflammation are often affected by joint effusion due to vascular leakage and synovial membrane edema (Palmer and Bertone, 1996). Mitigating the inflammatory response due to exercise load can reduce the potential for intra-articular tension and subsequently improve joint ROM. In the current study, plasma PGE_2_ concentrations were not influenced by dietary treatment; however, plasma PGE_2_ concentrations were reduced for both treatment groups in samples obtained 18 h after both exercise stressors were completed. Previous research comparing exercised and non-exercised horses has demonstrated a significant elevation in plasma PGE_2_ levels within 1-h postexercise stressor, with a concentration of 61 ± 1.8 pg/mL for exercising horses and 28 ± 13.1 pg/mL for the nonexercising group (MacNicol et al., 2018). These authors also demonstrated no significant difference in PGE_2_ concentrations between the groups from 2 to 24 h post-stressor; whereas the current trial detected an average decrease in PGE_2_ concentrations among all horses of 495.07 pg/mL during the first stressor and 289.12 pg/mL in the second. In a future study, increasing the frequency of sampling timepoints immediately after completion of the exercise stressor and assessing other markers of inflammation such as interleukin-1β and tumor necrosis factor-α may provide a more comprehensive and sensitive analysis of systemic inflammation and the impact of nutraceutical intervention (Goldring and Goldring, 2004; Watts et al., 2016).

In the current trial, dietary treatment did not affect type II cartilage catabolism or anabolism; however, mean serum C2C and CPII concentrations tended to increase over time. This change may be reflective of increased workload in horses’ daily exercise routine as a result of metabolic turnover. The C2C antibody recognizes neoepitopes generated by collagenase-cleaved type II collagen (Nicholson et al., 2010). The CPII marker, also termed “chondrocalcin,” is indicative of intra-articular cartilage that is undergoing repair as a result of pathological disturbance (McIlwraith, 1996). Additionally, CPII concentration will appear proportional to the rate of new collagen development. The half-life of CPII is relatively short (16 h) in synovial fluid, making it a useful biomarker of recent collagen synthesis (Garvican et al., 2010). Type II collagen is highly prevalent in joints and its catabolism is linked to OA progression (McIlwraith, 2005). When exposed to an intra-articular lipopolysaccharide inflammatory challenge, C2C and CPII concentrations in the synovial fluid have been shown to increase (275.1 ± 11.0 and 2891.2 ± 216.1 ng/mL, respectively) in mature horses, which suggests the potential for exercise-induced inflammation to initiate a similar response (Kahn et al., 2017). However, horses in the current study showed an average reduction of 364.71 ng/mL in CPII concentration 18 h after the day 13 exercise stressor, and no significant change in C2C concentration. In a study of collagen biomarkers in foals, horses with forced exercise showed lower concentrations of serum CPII (387.8 ± 26.2 ng/mL) than foals turned out on pasture, (522.4 ± 35.8 ng/mL), indicating the potential for a negative effect on type II collagen metabolism in the young exercised horse (Billinghurst et al., 2003).

Serum biomarkers can be useful in detecting intra-articular change once the protein has left the synovium and entered circulation (Ray et al., 1996). In the current study, no effect of dietary treatment was seen on serum Log_10_ CS846 concentrations; however, the supplement appeared to initiate a tendency for greater change from day 0 to 28 for horses on the TRT diet by 0.04 ± 0.05 ng/mL. These results are similar to observations from a prior study which recorded an increase in serum CS846 concentrations in healthy 2-yr-old horses from their baseline to day 7 of exercise (Frisbie et al., 2008). Another study reported no change in serum CS846 concentration among weanling horses undergoing sprint exercises (Billinghurst et al., 2003). A possible reason for these conflicting results may be age differences between the populations studied. The presence of the CS846 molecule reflects synthesis of intra-articular aggrecan molecules of the cartilage extracellular matrix, which are utilized in joint compression and shock absorption. The concentration of CS846 is highest in developing animals and declines with advancing age (Ray et al., 1996; Chavez, 2016). Also, elevated serum concentrations in horses may indicate articular cartilage turnover as a result of exercise (Ray et al., 1996; Leatherwood et al., 2018; MacNicol et al., 2018). Additionally, in the current study, the CS846 concentration tended to decrease in serum of all horses 18 h after completion of the second exercise stressor, from day 27 to d 28. These results contradict findings from Okumura et al. (2002), who observed the highest concentration of the cartilage marker keratan sulfate in the 30-min post-training period in 2- to 4-yr-old racehorses, and a return to baseline within 1 h after the conclusion of the training session.

Nutraceutical supplementation of glycosaminoglycans, sodium hyaluronate, glucosamine, and chondroitin are most frequently investigated as alternative methods to aid in joint recovery (Watts et al., 2016). Laverty et al. (2005) provided glucosamine through a nasogastric tube and reported a detectable level of the monosaccharide in synovial fluid up to 12-h postadministration, suggesting a potential for short-term pharmacokinetic impacts. In contrast to this study, Leatherwood et al. (2016) evaluated long-term oral supplementation of glucosamine and observed peak concentrations in synovial fluid at day 28 and 84 and in plasma at d 28 and 42 in yearling horses during a 98-d trial. The authors suggest that due to rapid metabolism of glucosamine, an extended period of supplementation may be required in order to stimulate a physiological effect due to the timeframe required for cartilage turnover. Incorporation of the supplement’s key ingredients into the intra-articular environment was not evaluated in the current study; however, it is possible that the 28-d supplementation period in the current study did not allow sufficient time for the cartilage in the horses’ joints to be altered.

A study utilizing a similar multi-ingredient joint supplement to the current study (MSM, HA, glucosamine, chondroitin sulfate) provided horses with the product for 61 d prior to conducting an intra-articular lipopolysaccharide challenge and observed significantly lower PGE_2_ concentrations in the treatment group compared with the placebo (van de Water et al., 2016). Unfortunately, authors did not evaluate PGE_2_ prior to d 28, so it is unknown if the ingredients would have elicited an effect earlier in the trial. In Watts et al. (2016), a resveratrol supplement was administered to horses after receiving triamcinolone acetonide injections in both hind tarsometatarsal joints. After 2 mo, owners administering the resveratrol reported improvement in horse performance at a significantly higher rate than owners administering the placebo. Furthermore, objective analysis using an inertial sensor indicated a significant improvement in the lameness amplitude: natural pelvic amplitude ratio among horses administered resveratrol compared with the control group (Watts et al., 2016).

Silicon, another ingredient included in the current study’s oral joint supplement, is highly prevalent in connective tissue and has shown promise as a beneficial supplement in reducing injury under extreme exercise stress in young racehorses (Carlisle, 1982; Nielsen et al., 1993). In Nielsen et al. (1993), 18-mo-old racehorses entering training were either not supplemented or received one of three different quantities of sodium zeolite A (**SZA**) for 180 d. Horses fed the highest dose at 2.8% SZA showed an increase in plasma silicon levels from 6.39 mg/dL on day 90 to 7.26 mg/dL on day 180. Additionally, horses fed SZA had faster race times and were able to race longer distances before reaching structural failure, suggesting that supplementation containing this ingredient may have a beneficial impact on performance and longevity in the performance horse. In contrast to this study, horses in the current study underwent light-exercise and had reached skeletal maturity, which may factor into the detectable impact of silicon in the TRT group.

In summary, an oral joint supplement was tested in a 28-d experiment using mature stock-type horses undergoing light exercise. Horses supplemented with TRT increased ROM of the hock at the walk and tended to increase ROM at the trot compared with horses in the CON group. These findings indicate that the hock may initially be the most sensitive to biomechanical change as a result of the nutraceutical supplementation. Furthermore, the hock is a common area sensitive to injury and affected by OA and supplementing this product may improve comfort and longevity in the mature horse. No change was observed in response to supplementation in serum or plasma biomarkers; however, further studies utilizing arthrocentesis, as well as examining additional markers for inflammation, may provide a more detailed understanding of the impact this supplement has intra-articularly in the mature horse. Testing this product in different populations, such as young horses entering performance training, and increasing the duration of supplementation prior to initiating a stressor, may also be useful to test the product’s ability to mitigate inflammation and joint degradation.

## References

[CIT0001] AAEP 2019 Lameness exams: Evaluating the lame horse. American Association of Equine Practitioners, Web Link https://aaep.org/horsehealth/lameness-exams-evaluating-lame-horse

[CIT0002] Abramson S B 2008 Osteoarthritis and nitric oxide. Osteoarthr Cartilage. 16(Suppl 2):S15–S20. doi:10.1016/S1063-4584(08)60008-4. 18794013

[CIT0003] Attur M, Statnikov A, Aliferis C F, Li Z, Krasnokutsky S, Samuels J, Greenberg J D, Patel J, Oh C, Lu Q A, and Ramirez R. 2012 Inflammatory genomic and plasma biomarkers predict progression of symptomatic knee OA (SKOA). Osteoarthr Cartilage. 20:S34–S35. doi: 10.1016/j.joca.2012.02.562 PMC4630783 26521737

[CIT0004] Billinghurst R C, Brama P A, van Weeren P R, Knowlton M S, and McIlwraith C W. 2003 Significant exercise-related changes in the serum levels of two biomarkers of collagen metabolism in young horses. Osteoarthr Cartilage. 11:760–769. doi:10.1016/s1063-4584(03)00152-3. 13129696

[CIT0005] Cantley C E, Firth E C, Delahunt J W, Pfeiffer D U, and Thompson K G. 1999 Naturally occurring osteoarthritis in the metacarpophalangeal joints of wild horses. Equine Vet. J. 31:73–81. doi:10.1111/j.2042-3306.1999.tb03794.x. 9952333

[CIT0006] Carlisle E M 1982 The nutritional essentiality of silicon. Nutr. Rev. 40:193–198. doi:10.1111/j.1753-4887.1982.tb05307.x. 6750461

[CIT0007] Chateau H, Holden L, Robin D, Falala S, Pourcelot P, Estoup P, Denoix J M, and Crevier-Denoix N. 2010 Biomechanical analysis of hoof landing and stride parameters in harness trotter horses running on different tracks of a sand beach (from wet to dry) and on an asphalt road. Equine. Vet. J. 42: 488–495. doi:10.1111/j.2042-3306.2010.00277.x 21059050

[CIT0008] Chavez H, Folch H, Araya O, Uberti B, and Moran G. 2016 Concentration of the CS-846 epitope in serum and synovial fluid of horses with different grades of osteochondral fragments in the carpal joints. General Medicine Open Access (Los Angeles) 4(3): 1–5. doi:10.4172/2327-5146.1000242

[CIT0009] Clayton H M 1991a Advances in motion analysis. Vet. Clin. North Am. Equine Pract. 7:365–382. doi:10.1016/s0749-0739(17)30504-7. 1933568

[CIT0010] Clayton H M 1991b Conditioning sport horses. Mason (OH): Sport Horse Publications.

[CIT0011] Clayton H M, Almeida P E, Prades M, Brown J, Tessier C, and Lanovaz J L. 2002 Double-blind study of the effects of an oral supplement intended to support joint health in horses with tarsal degenerative joint disease. Proc. Ann. Convention AAEP. 48:314–317.

[CIT0012] Clayton H M, and van Weeren P R. 2013 Performance in equestrian sports. Equine Locomotion, 2nd edition. St. Louis (MO): Saunders Elsevier.

[CIT0013] Coverdale J A, and Campbell J M. 2014 Administration of bioactive proteins to mature horses improves gait kinematics. J. Anim. Sci. 92(E-Supple. 2):599.

[CIT0014] Coverdale J A, and Campbell J M. 2015 Influence of bioactive proteins in varying doses on gait kinematics in mature horses. J. Eq. Vet. Sci. 35(5): 416. doi:10.1016/j.jevs.2015.03.087

[CIT0015] Forsyth R K, Brigden C V, and Northrop A J. 2006 Double blind investigation of the effects of oral supplementation of combined glucosamine hydrochloride (GHCL) and chondroitin sulfate (CS) on stride characteristics of veteran horses. Equine Vet. J. 38(S36): 622–625. doi:10.1111/j.2042-3306.2006.tb05615.x 17402494

[CIT0016] Frisbie D D, Al-Sobayil F, Billinghurst R C, Kawcak C E, and McIlwraith C W. 2008 Changes in synovial fluid and serum biomarkers with exercise and early osteoarthritis in horses. Osteoarthr Cartilage. 16:1196–1204. doi:10.1016/j.joca.2008.03.008. 18442931

[CIT0017] Galisteo A M, Cano M R, Morales J L, Miro F, Vivo J, and Agüera E. 1997 Kinematics in horses at the trot before and after an induced forelimb supporting lameness. Equine Vet. J. 29(S23): 97–101. doi:10.1111/j.2042-3306.1997.tb05064.x 9354300

[CIT0018] Garvican E R, Vaughan-Thomas A, Innes J F, and Clegg P D. 2010 Biomarkers of cartilage turnover. Part 1: markers of collagen degradation and synthesis. Vet. J. 185:36–42. doi:10.1016/j.tvjl.2010.04.011. 20488735

[CIT0019] Godges J J, MacRae P G, and Engelke K A. 1993 Effects of exercise on hip range of motion, trunk muscle performance, and gait economy. Phys. Ther. 73:468–477. doi:10.1093/ptj/73.7.468. 8316580

[CIT0020] Goldring S R, and Goldring M B. 2004 The role of cytokines in cartilage matrix degeneration in osteoarthritis. Clin. Orthop. Relat. Res. 427:S27–36. doi:10.1097/01.blo.0000144854.66565.8f 15480070

[CIT0021] Hanson R R, Smalley L R, Huff G K, White S, and Hammad T A. 1997 Oral treatment with a glucosamine-chondroitin sulfate compound for degenerative joint disease in horses: 25 cases. Eq. Prac. (19):16–22.

[CIT0022] Hewetson M, Christley R M, Hunt I D, and Voute L C. 2006 Investigations of the reliability of observational gait analysis for the assessment of lameness in horses. Vet. Rec. 158:852–857. doi:10.1136/vr.158.25.852. 16798953

[CIT0023] Kahn M K, Coverdale J A, Leatherwood J L, Arnold C E, Dabareiner R A, Bradbery A N, Millican A A, and Welsh T H. 2017 Age-related effects on markers of inflammation and cartilage metabolism in response to an intra-articular lipopolysaccharide challenge in horses. J. Anim. Sci. 95:671–680. doi:10.2527/jas.2016.1078. 28380609

[CIT0555] Laverty S, Sandy J D, Celeste C, Vachon P, Marier J F, and Plaas A H. 2005 Synovial fluid levels and serum pharmacokinetics in a large animal model following treatment with oral glucosamine at clinically relevant doses. Arthritis & Rheumatism: Official Journal of the American College of Rheumatology. 52(1):181–191. doi:10.1002/art.20762. 15641100

[CIT0024] Leatherwood J L, Coverdale J A, Arnold C E, and Scott B D. 2018 Effect of n-3 polyunsaturated fatty acids on markers of inflammation in young horses in training. Prof. Anim. Sci. 34(3): 284–292. doi:10.1093/jas/skx076

[CIT0025] Leatherwood J L, Gehl K L, Coverdale J A, Arnold C E, Dabareiner R A, Walter K N, and Lamprecht E D. 2016 Influence of oral glucosamine supplementation in young horses challenged with intra-articular lipopolysaccharide. J. Anim. Sci. 94:3294–3302. doi:10.2527/jas.2016-0343. 27695773

[CIT0026] MacNicol J L, Lindinger M I, and Pearson W. 2018 A time-course evaluation of inflammatory and oxidative markers following high-intensity exercise in horses: a pilot study. J. Appl. Physiol. (1985). 124:860–865. doi:10.1152/japplphysiol.00461.2017. 29074709 PMC5972457

[CIT0027] McIlwraith C W 1996 Chapter 3: General pathobiology of the joint and response to injury. In: McIlwraith C W and Trotter G W, editors, Joint disease in the horse. Philadelphia (PA): W. B. Saunders Co, pp. 104–117.

[CIT0028] McIlwraith C W 2005 Use of synovial fluid and serum biomarkers in equine bone and joint disease: a review. Equine Vet. J. 37:473–482. doi:10.2746/042516405774480102. 16163952

[CIT0029] McIlwraith C W 2008 Use of synovial fluid and serum biomarkers in equine bone and joint disease: a review. Equine Musculoskeletal Biomarkers. 37(5): 1–5. doi:10.2746/042516405774480102 16163952

[CIT0031] Morales J L, Manchado M, Vivo J, Galisteo A M, Aguera E, and Miro F. 1998 Angular kinematic patterns of limbs in elite and riding horses at the trot. Equine Vet. J. 30(6): 528–533. doi:10.1111/j.2042-3306.1998.tb04529.x 9844972

[CIT0032] Nicholson A M, Trumble T N, Merritt K A, and Brown M P. 2010 Associations of horse age, joint type, and osteochondral injury with serum and synovial fluid concentrations of type II collagen biomarkers in Thoroughbreds. Am. J. Vet. Res. 71:741–749. doi:10.2460/ajvr.71.7.741. 20594075

[CIT0033] Nielsen B D, Potter G D, Morris E L, Odom T W, Senor D M, Reynolds J A, Smith W B, Martin M T, and Bird E H. 1993 Training distance to failure in young racing quarter horses fed sodium zeolite A. J. Equine Vet. Sci. 13(10):562–567. doi:10.1016/S0737-0806(06)81526-1

[CIT0034] NRC 2007 Nutrient requirements of horses. 6th rev. ed. Washington (DC): National Academic Press.

[CIT0035] Okumura M, Kim G H, Tagami M, Haramaki S, and Fujinaga T. 2002 Serum keratan sulphate as a cartilage metabolic marker in horses: the effect of exercise. J. Vet. Med. A. Physiol. Pathol. Clin. Med. 49:195–197. doi:10.1046/j.1439-0442.2002.00434.x. 12069261

[CIT0036] Palmer J L, and Bertone A L. 1996 Chapter 7: Joint biomechanics in the pathogenesis of traumatic arthritis. In: McIlwraith C W, and Trotter G W, editors, Joint disease in the horse. Philadelphia (PA): W.B. Saunders Co, pp. 104–117.

[CIT0037] Peham C, Licka T, Girtler D, and Scheidl M. 2001 The influence of lameness on equine stride length consistency. Vet. J. 162:153–157. doi:10.1053/tvjl.2001.0593. 11531399

[CIT0038] Ray C S, Poole A R, and McIlwraith C W. 1996 Use of synovial fluid and serum markers in articular disease. In: McIlwraith C W, and Trotter G W, editors, Joint disease in the horse. Philadelphia (PA): W.B. Saunders Co, pp. 203–216.

[CIT0039] Tessutti V, Ribeiro A P, Trombini-Souza F, and Sacco I C. 2012 Attenuation of foot pressure during running on four different surfaces: asphalt, concrete, rubber, and natural grass. J. Sports Sci. 30:1545–1550. doi:10.1080/02640414.2012.713975. 22897427

[CIT0444] Van de Water E, Oosterlinck M, Dumoulin M, Korthagen N M, van Weeren P R, Van den Broek J, Everts H, Pille F, and van Doorn D A. 2017 The preventive effects of two nutraceuticals on experimentally induced acute synovitis. Equine Vet. J. 49(4):532–538. doi:10.1111/evj.12629. 27554764 PMC5484312

[CIT0040] Watts A E, Dabareiner R, Marsh C, Carter G K, and Cummings K J. 2016 A randomized, controlled trial of the effects of resveratrol administration in performance horses with lameness localized to the distal tarsal joints. J. Am. Vet. Med. Assoc. 249:650–659. doi:10.2460/javma.249.6.650. 27585103

[CIT0041] Woodward A D, Nielsen B D, O’Connor C I, Webel S K, and Orth M W. 2005 Dietary long chain polyunsaturated fatty acids increase plasma eicosapentaenoic acid and docohexaenoic acid concentrations and trot stride length in horses. In: Proceedings of the 19th Equine Science Society Symposium, May 31-June 3, Tucson, Arizona. pp. 101–106.

[CIT0042] Yamanobe A, Hiraga A, and Kubo K. 1993 Relationships between stride frequency, stride length, step length and velocity with asymmetric gaits in the Thoroughbred horse. Japanese J. Equine Sci. 3(2):143–148. doi:10.1294/jes1990.3.143

